# Evaluation RNAi silencing in the DH82 canine histiocytic sarcoma cell line

**DOI:** 10.1186/1753-6561-8-S4-P158

**Published:** 2014-10-01

**Authors:** Gabriel Silva, Natália Yumi Noronha, Tatiana Takahasi Komoto, Ana Lúcia Fachin, Mozart Marins

**Affiliations:** 1 Universidade de Ribeirão Preto, Ribeirão Preto, SP, Brazil

## Background

Cancer is a leading cause of death among dogs worldwide [1]. The disease results from alterations in expression of genes that control the cell cycle, including proliferation, differentiation and programmed cell death [2]. The interest in understanding the molecular aspects of cancer in humans and dogs is driven by the possibilities of identification of novel targets for the development of new anticancer compounds [3]. A molecular tool that has been used for this purpose is the RNA interference (RNAi) technique which exploits the mechanism of post-transcriptional silencing, mediated by molecules of double-stranded RNA that trigger degradation of complementary mRNAs [4]. However, for the application of this technique, important aspects must be investigated, since not all cell strains are susceptible to silencing by RNAi, and often the intracellular release of the interfering RNAs has low efficiency [5]. In this work we evaluated the efficiency of RNAi in silencing of topoisomerase IIA, a target for many anticancer compounds, in the canine histiocytic sarcoma DH82 cell line.

## Materials and methods

DH82 cells at a concentration of 2,4 × 10^5 ^cells/well were cultured in 6 and 24 well plates at 37°C and 5% CO_2_. Next, siRNAs (small interfering RNA) designed to target topoisomerase IIA and glyceraldehyde-3-phosphate dehydrogenase (positive control) mRNA were introduced into cells with the kit N-TER Nanoparticle siRNA Transfection System (Sigma Aldrich^®^). The cells were incubated for 24 hours with a solution of nanoparticles, comprising siRNA (20-30 nM) complexed to peptides N-TER. After transfection, the silencing was confirmed by real time PCR using TaqMan Gene Expression Assays (Applied Biosystems^®^). The ΔΔCt method was used for calculating differences in gene expression levels between the untransfected and transfected cells. The level of expression is shown as a fold change positive (induction) or negative (repression).

## Results and discussions

Silencing of topoisomerase IIA (top IIA) and glyceraldehyde-3-phosphate dehydrogenase (GAPDH) was confirmed by obtaining negative fold change. Following transfection in 6-well plates, it was obtained a fold change of -2,56 and -1,35 for the top IIA and GAPDH genes, respectively. The level of silencing of type IIA decreases after incubation of the transfected cells in the absence of siRNA, as demonstrated by reduced values of the fold change for -0,95 and -0,82 after 24 and 48 hours of incubation, respectively. The transfection in 6 and 24 well plates resulted in silencing of the top IIA of -2,56 and -0,73. The use of 24 well plate for transfection, possibly facilitates the internalization of siRNA/peptide complex by cells. The increased concentration of siRNA from 20 to 30 nM did not result in increased silencing of the top IIA.

## Conclusions

The lineage DH82 demonstrated susceptibility to silencing mediated by RNAi. The use of RNAi technique in this cell lineage when performed under ideal conditions of transfection was efficient, allowing the use of this model as a tool to assist in the identification of new targets for the development of new anticancer drugs.

## Financial support

This work was supported for CAPES and FAPESP.
